# Chemonucleolysis combined with dynamic loading for inducing degeneration in bovine caudal intervertebral discs

**DOI:** 10.3389/fbioe.2023.1178938

**Published:** 2023-08-30

**Authors:** Andrea Vernengo, Helen Bumann, Nadine Kluser, Astrid Soubrier, Amra Šećerović, Jan Gewiess, Jan Ulrich Jansen, Cornelia Neidlinger-Wilke, Hans-Joachim Wilke, Sibylle Grad

**Affiliations:** ^1^ AO Research Institute Davos, Davos, Switzerland; ^2^ Institute of Orthopaedic Research and Biomechanics, Ulm University, Ulm, Germany; ^3^ Department of Health Sciences and Technology, ETH Zürich, Zurich, Switzerland

**Keywords:** organ culture, chemonucleolysis (CN), intervertebral disc, degeneration, extracellular matrix

## Abstract

Chemonucleolysis has become an established method of producing whole organ culture models of intervertebral disc (IVD) degeneration. However, the field needs more side-by-side comparisons of the degenerative effects of the major enzymes used in chemonucleolysis towards gaining a greater understanding of how these organ culture models mimic the wide spectrum of characteristics observed in human degeneration. In the current work we induced chemonucleolysis in bovine coccygeal IVDs with 100 µL of papain (65 U/mL), chondroitinase ABC (chABC, 5 U/mL), or collagenase II (col’ase, 0.5 U/mL). Each enzyme was applied in a concentration projected to produce moderate levels of degeneration. After 7 days of culture with daily dynamic physiological loading (0.02–0.2 MPa, 0.2 Hz, 2 h), the cellular, biochemical and histological properties of the IVDs were evaluated in comparison to a PBS-injected control. Papain and collagenase, but not chABC, produced macroscopic voids in the tissues. Compared to day 0 intact IVDs, papain induced the greatest magnitude glycosaminoglycan (GAG) loss compared to chABC and col’ase. Papain also induced the greatest height loss (3%), compared to 0.7%, 1.2% and 0.4% for chABC, col’ase, and PBS, respectively. Cell viability in the region adjacent to papain and PBS-injection remained at nearly 100% over the 7-day culture period, whereas it was reduced to 60%–70% by chABC and col’ase. Generally, enzyme treatment tended to downregulate gene expression for major ECM markers, type I collagen (COL1), type II collagen (COL2), and aggrecan (ACAN) in the tissue adjacent to injection. However, chABC treatment induced an increase in COL2 gene expression, which was significant compared to the papain treated group. In general, papain and col’ase treatment tended to recapitulate aspects of advanced IVD degeneration, whereas chABC treatment captured aspects of early-stage degeneration. Chemonucleolysis of whole bovine IVDs is a useful tool providing researchers with a robust spectrum of degenerative changes and can be utilized for examination of therapeutic interventions.

## Introduction

Degeneration of the lumbar intervertebral disc (IVD) is a frequent medical occurrence. Its clinical presentation is diverse, varying from asymptomatic patients whose lumbar spines show radiological degenerative changes up to severe chronic pain (Brinjikji et al., 2015). Multiple studies have shown a close association between degeneration of the IVD and low back pain (LBP) (Paajanen et al., 1997; de Schepper et al., 2010; Rahyussalim et al., 2020). To date, LBP caused by IVD degeneration is mainly treated by the administration of pain medication, physiotherapy, or surgery. These treatment options target the reduction of pain without directly addressing the root cause, and thus have not been proven effective for reversing disease progression. There is an urgent clinical need for improved treatments which are aimed at restoring the structure and function of IVD tissue.

The IVD consists of connective tissue bridging two vertebrae with each other, therefore allowing motion between the osseous parts of the spine (Bogduk, 2016). The IVD is a composite structure comprised of a nucleus pulposus (NP), annulus fibrosus (AF), and cartilaginous endplates (CEP) (Dowdell et al., 2017). It has been proposed that degeneration is initiated in the NP region (Barcellona et al., 2022), due to numerous possible factors such as calcification, microvascular disease, or smoking (Daly et al., 2016). The reported activity of IVD cells during degeneration has been variable, marked by cell death (Liao et al., 2019; Yang et al., 2022), increased proliferation and cluster formation (Lama et al., 2019; Bonnaire et al., 2021), or senescence (Zhang et al., 2020a; Song et al., 2023). The tissue matrix content in degenerative IVDs is characterized by a continuous loss of proteoglycans (PGs) and water content from NP and inner AF (iAF) tissue (Urban and Roberts, 2003; Freemont, 2009). In the early stages of degeneration, COL2 content increases in the NP, possibly as a salvage attempt by the cells (Takaishi et al., 1997; Cs-Szabo et al., 2002; Uei et al., 2006; Zhou et al., 2021). However, as degeneration advances, COL2 is replaced by COL1, and concomitantly increased levels of COL2 are observed in the outer AF (oAF) (Le Maitre et al., 2007a). Degeneration is further characterized by the elevated expression of proinflammatory cytokines such as IL-1β (Le Maitre et al., 2007b) and IL-8 (Ahn et al., 2002), as well as catabolic enzymes such as matrix metalloproteinases (MMPs) (Roberts et al., 2000; Liu et al., 2018) and the A Disintegrin and Metalloproteinase with Thrombospondin motifs (ADAMTS) family of enzymes (Wang et al., 2015; Wang et al., 2017). Taken together, these alterations lead to a shift in the ECM composition at the center of the IVD, driving abnormal biomechanics and leading to the appearance of fissures in the AF, NP herniation, and overall disc height loss (Boos et al., 2002). However, the transcriptomic and proteomic profiles of an IVD during degeneration can depend on anatomical location (Panebianco et al., 2021; van den Akker et al., 2017), age (Nerlich et al., 2007), genetics (Miller et al., 1988), inflammatory signals (Zhang et al., 2021), and mechanical stress/loading patterns (Wang et al., 2020; Fu et al., 2021). Collectively, research indicates that there will be no “one size fits all” cure for reversing IVD degeneration, and that discerning effective therapeutic interventions will require testing platforms that can mimic the wide spectrum of characteristics observed in degenerating IVDs.

To date, various interventions such as growth factors (Zhu et al., 2019; Kim et al., 2020), cell-based therapies (Panebianco et al., 2020; Barcellona et al., 2021), and injectable biomaterials (Pennicooke et al., 2018; Fujii et al., 2020) have been explored towards achieving tissue reparation. For evaluating the efficacy of these approaches, several *in vitro*, *ex vivo* and *in vivo* models of IVD degeneration have been developed, each with its own set of limitations. *In vitro* models generally require isolation of IVD cells from their native tissues, making the outcomes less predictive of the clinical situation (Teixeira et al., 2020). Meanwhile, *in vivo* models are more predictive, but they are costly, time-intensive and have ethical disadvantages. *Ex vivo* organ models serve as cost-effective testing platforms bridging *in vitro* and *in vivo* studies (Alini et al., 2008). In recent years, a number of whole IVD explant organ culture systems have been developed, mainly focused on bovine coccygeal discs due to their similarity to human lumbar vertebrae in terms of cellular activity, tissue composition and geometry (Oshima et al., 1993; Alini et al., 2008). In such models, fresh tissue explants are derived from recently slaughtered animals and degeneration can be induced by a variety of methods, such as mechanical overload (Christiani et al., 2021; Fu et al., 2021), proinflammatory cytokines (Du et al., 2020), structural injury (Korecki et al., 2008), or chemonucleolysis (Roberts et al., 2008; Gullbrand et al., 2017; Rustenburg et al., 2020).

Chemonucleolysis involves the intradiscal delivery of enzymes to induce cleavage and release of native ECM components, thus initiating a degenerative cascade. At present, a variety of *in vivo* and *ex vivo* IVD degeneration models using chemonucleolysis have been established. For instance, chondroitinase ABC (chABC) has been used to induce degradation of glycosaminoglycans (GAG) side chains of PGs, leading to decreased disc height, loss of structural integrity, and increased expression of inflammatory mediators (Imai et al., 2007; Gullbrand et al., 2017; Zhang et al., 2020b). Other studies with IVD chemonucleolysis have utilized papain, a plant proteolytic enzyme that cleaves peptide bonds in basic amino acids (Mamboya, 2012). Papain was initially used as a therapy in IVD herniation (Javid, 1980), but was later studied *ex vivo*, inducing losses of GAG, disrupted structure of the tissue, and degenerative biomechanical properties (Uei et al., 2006; Roberts et al., 2008; Chan et al., 2013). Collagenase enzymes have the ability to degrade triple-helical native collagen fibrils, (Van Wart and Steinbrink, 1985), leading to significant IVD height loss *in vivo* 2–7 days post-injection (Kalaf et al., 2014) and osteophyte formation at 3 months (Stern and Coulson, 1976). *Ex vivo,* collagenase treatment increased expression of inflammatory cytokines, such as IL-1β, and matrix changes consistent with moderate human IVD degeneration (Rustenburg et al., 2020). Importantly, multiple studies show that injecting higher concentrations of matrix-degrading enzymes produces more aggressive degenerative changes (Chan et al., 2013; Gullbrand et al., 2017; Rustenburg et al., 2020). However, due to the aforementioned complexities of human IVD degeneration, it is unlikely that any single model can effectively recapitulate the disease. In fact, to increase the quality of new reparative technologies, the field needs to continue exploring models of IVD degeneration in order to effectively capture the wide breadth of phenotypic and molecular changes associated with the disease. While chemonucleolysis models have potential to be very useful in this respect, to date there has been no side-by-side comparisons of the commonly used enzymes to elucidate similarities and differences in their degenerative signatures across the NP and AF of an IVD.

To this end, in the current work we induced chemonucleolysis in bovine coccyegeal IVDs with injection of three different enzymes, papain, chABC, and collagenase. It was hypothesized that each of the enzymes, having a unique substrate in the IVD ECM, would induce a distinct subset of degenerative characteristics in the tissues. Comprising this study, the enzymes were applied in a concentration projected to produce moderate levels of degeneration based on earlier observations (Chan et al., 2013; Gullbrand et al., 2017; Rustenburg et al., 2020). The resulting cellular, biochemical and histological properties of the IVDs were evaluated adjacent to the site of enzyme injection (the iAF) and in the oAF. From a broader perspective, we posited the varied spectrum of degenerative responses would provide multiple viable platforms mimicking known degenerative responses in IVD tissues and allowing for more thorough evaluation of pre-clinical therapeutics.

## Materials and methods

### Dissection of bovine IVD

Caudal IVDs from bovine tails were collected fresh from a local slaughterhouse (all male, age less than 24 months, and a minimum of five IVDs were isolated per tail). IVDs were isolated from the tails with endplates (EP) and a minimum amount of bone from their surrounding tissue as described previously (Lang et al., 2018). The IVDs were cleaned via a Pulsavac wound debridement irrigation system (Zimmer, Inc., Winterthur, Switzerland). After isolation, except for IVDs set aside as day 0 intact samples, the remainder of the samples were cultured in a 6-well-plate with 8 mL of complete IVD culture medium containing high glucose Dulbecco’s modified Eagle’s medium (HG DMEM) supplemented with 2.5% HEPES buffer (Gibco), 1% Penicillin/Streptomycin (Gibco), ascorbate 2 phosphate (50 ug/mL, Sigma Aldrich), 1% ITS+ (Corning), 2% fetal calf serum, 1% non-essential amino acid (Gibco) and 50 ug/mL primocin (Invitrogen). The IVD specimens were cultured at 37°C and 5% CO_2_ for a total of 7 days. Cultured samples were transferred daily to a bioreactor for dynamic axial loading applied between 0.02 and 0.2 MPa at a frequency of 0.2 Hz for 2 h, a regime established in previous work to be optimal for maintenance of cell metabolic activity (Maclean et al., 2004; Gantenbein et al., 2006; Paul et al., 2012; Lang et al., 2018). Each experimental group consisted of *n* = 4 replicates isolated from two to four donors.

### Enzyme treatment

**FIGURE 1:**
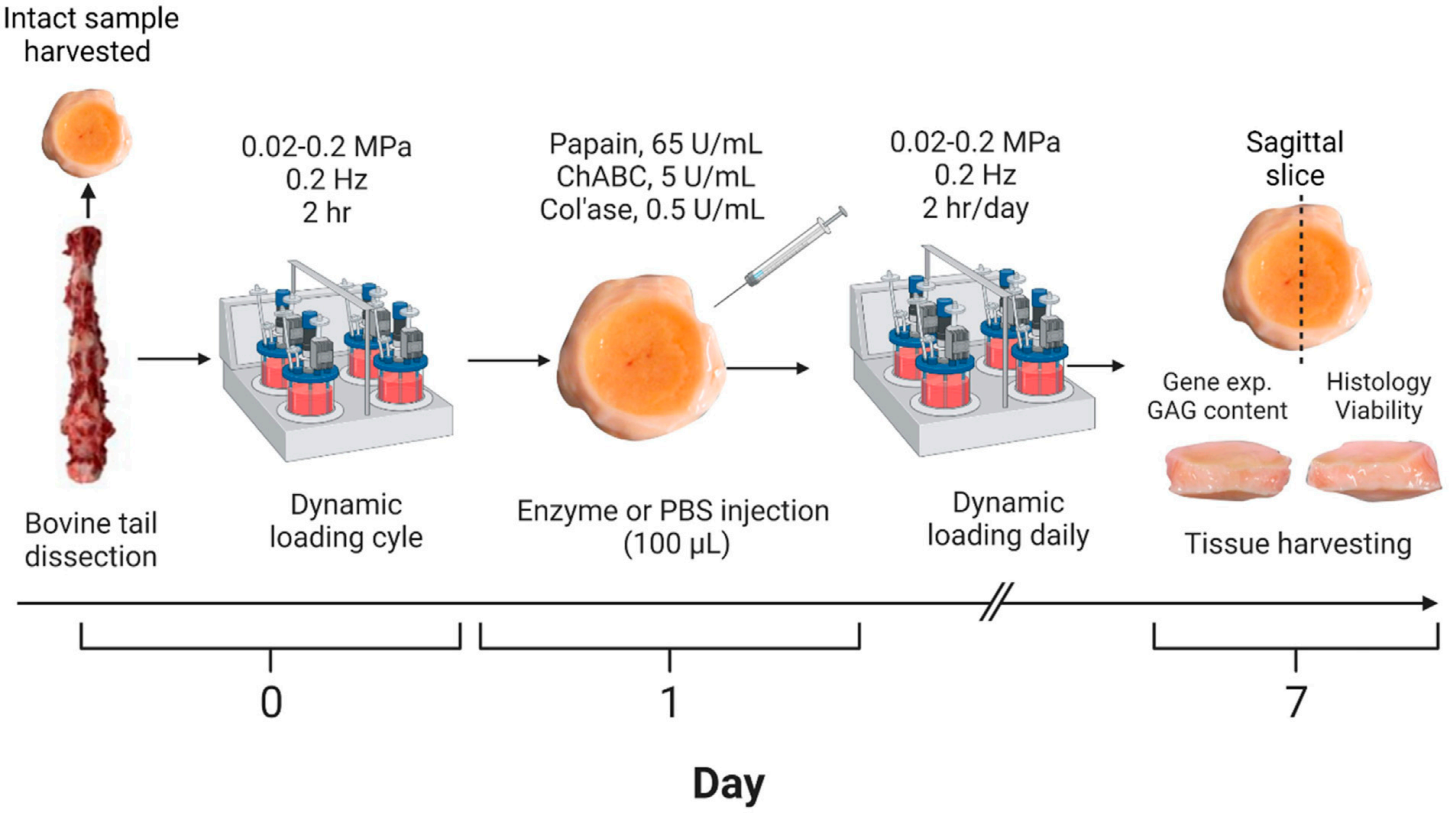
Workflow for developing the enzyme-induced model of IVD degeneration. Bovine coccygeal IVDs were isolated. An intact IVD from each tail was set aside as the day 0 control, and the remaining samples subjected to dynamic axial loading. The IVDs were subsequently injected with 100 µL of PBS (control), papain (65 U/mL), chABC (5 U/mL) or col’ase (0.5 U/mL) and cultured for 7 days with daily physiological loading applied. IVD height was measured daily prior to each loading cycle. At the end of the study period, the specimens were sliced along the sagittal plane and evaluated for gene expression, GAG content, histological staining, and cell viability analyses. A total of *n* = 4 replicates were used per enzyme/control group, isolated from two to four donors.

Papain (from *Carica papaya*, Roche) was diluted to a concentration of 65 U/mL (Chan et al., 2013) in phosphate buffered saline (PBS) containing 0.8 mM cysteine HCl and 0.4 mM Ethylenediaminetetraacetic acid (EDTA). The chondroitinase ABC suspension (chABC, from *proteus* vulgaris, Sigma Aldrich) was delivered at a concentration of 5 U/mL in PBS containing 0.1% bovine serum albumin (BSA) (Gullbrand et al., 2017). Collagenase II (col’ase, Gibco) was prepared at a concentration of 0.5 U/mL (Rustenburg et al., 2020) in Hank’s salt balanced solution. On day 1 immediately after the first application of loading, the isolated IVDs were randomly assigned to receive either PBS (*n* = 4) or enzyme injection (*n* = 4 per enzyme). Enzyme suspensions (100 µL) were manually injected into the center of each IVD using an insulin syringe (29G). All results were compared to the intact tissue samples from the respective tail (day 0) that were never cultured. The general study design is summarized in Figure 1.

### Disc height measurement

Disc height was measured immediately after dissection (day 0) and daily for 7 days immediately prior to loading. Each disc was measured with calipers at two positions and the average value was used to calculate disc height change at day 7 normalized to the initial height after dissection.

### Histological analysis

At day 7 of culture the IVDs were harvested and cut into two equal halves along the sagittal plane. One half was taken for histological and cell viability analyses, where the bony and cartilaginous end plates were removed prior to snap freezing in cryocompound. Sagittal sections (10 µm thick) were made with a cryotome (NX70 model; Thermo Fisher Scientific, Waltham, MA, United States). Representative sections from each group were stained with lactate dehydrogenase (LDH) and ethidium homodimer as previously described (Stoddart et al., 2006) to determine cell viability. For cell viability analyses, four random regions of interest (ROI) were analyzed in each the inner or outer annulus fibrosus (AF) regions across three representative sections per replicate and per group. Cells stained blue and blue/red were assigned to living cells, and cells that stained red were assigned to dead cells. The numbers of alive and dead cells were counted using ImageJ and expressed as a measure of cell viability per ROI. The cell viability in each region at day 7 was normalized to day 0 for the same donor.

Remaining histological sections were fixed in methanol and stained with 0.1% Safranin-O and 0.02% Fast Green and Weigert’s Haematoxylin to highlight proteoglycan, collagen, and cell nuclei, respectively. All sections were imaged with light microscope (Zeiss, Oberkochen, Germany and Olympus, Tokio, Japan) under transmitted and/or fluorescent light.

A semi-quantitative scoring scheme (Table 1) adapted from Lee et al. (2021) was used to evaluate the Safranin O/Fast Green staining. NP and AF structure and matrix characteristics were summed up by two blinded observers to assess the degree of IVD degeneration observed for day 0 intact, PBS, and enzyme-injected groups (*n* = 3 sections per group).

**TABLE 1:** Histological grading criteria, based on Lee et al. (2021).

Grade	Histological characteristics
NP matrix staining	
0	Proteoglycan staining dominates
1	Slight reduction in proteoglycan (fading)
2	Severe reduction in proteoglycan
3	Loss of proteoglycan staining
AF Morphology	
0	Well-organized, well-defined, uniform collagen lamellae form concentric half-ring arcs throughout entire AF
1	Mild disorganization/delamination of collagen fiber lamellae with some disruption or loss of concentric layers (<25%)
2	Moderately disorganization/delamination of collagen fiber lamellae with progressive disruption or loss of concentric layer (25%–75%)
3	Complete disorganization/delamination/collapse of AF; almost all concentric collagen lamellae are severely disrupted or lost (>75%)
Distinction between NP and AF	
0	Clear distinction between AF and NP tissue with intense purple proteoglycan ECM staining in NP
1	Distinction less clear: loss of annular-nuclear demarcation
2	Distinction poor: loss of annular-nuclear demarcation
3	No discernable annular-nuclear demarcation

### GAG content

The second half of the harvested IVDs was designated for quantitative analyses. Because the NP was digested by enzymes in many samples, the region closest to the enzyme-induced void was taken for analyses and designated as inner annulus fibrosus (iAF). In addition, tissue was sampled from the periphery of the IVDs, designated as outer annulus fibrosus (oAF). For GAG analysis, tissue samples from each IVD region (30–50 mg) were lyophilized and digested in 2 mL of 0.5 mg/mL proteinase K solution (Roche, Mannheim, Germany) per 10 mg of dry tissue until complete breakdown. Sulfated GAG content was measured by using 1,9-Dimethyl-methylene blue (Aldrich). Absorbance was read at 535 mm with a Victor3 Micro Plate Reader. GAG concentrations were calculated from a standard curve obtained with chondroitin 4-sulfate sodium salt from bovine trachea (Fluka Bio Chemika). GAG contents were normalized to initial wet tissue mass and to the GAG content of the day 0 tissue sample from the same donor.

### Gene expression

Tissue samples (100–200 mg) were cut from the iAF and oAF of the specimens. The oAF was defined in this study as the outermost fibrous ring of tissue on the specimens. Tissues were digested in 2 mg/mL pronase (Roche), snap frozen in liquid nitrogen and pulverized into powder pellets using a hammering device (Caprez et al., 2018). Pulverized samples were homogenized in 1.5 mL TRI reagent containing 7.5 µL polyacryl carrier (Molecular Research Centre Inc., Cincinnati, OH, United States) using a tissue-lyser (Retsch GmbH & Co., Haan, Germany). RNA was extracted with RNeasy columns (Qiagen) according to the manufacturer’s protocol. Reverse transcription was performed using SuperScript^®^ VILO™ cDNA Synthesis Kit (Invitrogen) with 400 ng total RNA according to the manufacturer’s protocol. QPCR was performed using TAQMAN™ Universal MasterMix (Applied Biosysems). The forward primer, reverse primer, probe (or Gene Expression Assay mixture, Applied Biosystems), and cDNA were combined to make a reaction volume of 10 µL. Gene expression for the following bovine transcripts were analyzed: RPLP0 (Ribosomal Protein Lateral Stalk Subunit P0, endogenous reference gene), TAGLN (transgelin), ELN (elastin), MKX (mohawk homeobox), MCAM (Melanoma Cell Adhesion Molecule), COL1 (collagen1α2), COL2 (collagen2α1), MMP3 (matrix metalloproteinase-3), ADAMTS5 (ADAM Metallopeptidase With Thrombospondin Type 1 Motif 5), ACAN (aggrecan), IL-1β (Interleukin-1beta), and IL-8 (CXCL8 Gene—C-X-C Motif Chemokine Ligand 8). All primer sequences or IDs of Gene Expression Assays are listed in Table 2. The qPCR was performed using QuantStudio 7 Flex (Applied Biosystems, Thermo Fisher Scientific) for 40 cycles with the following protocol: heating of 1.9°C per second until 95°C followed by 15 s at 95°C and cooling of 1.6°C/s to 60°C and continuing for 60 s at 60°C. The relative expression of analyzed genes was identified using 2^−ΔΔCt^ method with RPLP0 and day 0 expression from the same tissue donor used for normalization.

**TABLE 2:** Oligonucleotide primers (900 nM final concentration), probes (250 nM final concentration) (bovine) and gene expression assays (Applied Biosystems) used for quantitative real-time PCR.

Gene	Primer/Probe type sequence or assay ID	
ACAN	Primer forward (5′-3′)	5′-CCA ACG AAA CCT ATG ACG TGT ACT-3′
	Primer reverse (5′-3′)	5′-GCA CTC GTT GGC TGC CTC-3′
	Probe (5′FAM/3′TAMRA)	5′-ATG TTG CAT AGA AGA CCT CGC CCT CCA T-3′
COL1 A2	Primer forward (5′-3′)	5′-TGC AGT AAC TTC GTG CCT AGC A-3′
	Primer reverse (5′-3′)	5′-CGC GTG GTC CTC TAT CTC CA-3′
	Probe (5′FAM/3′TAMRA)	5′-CAT GCC AAT CCT TAC AAG AGG CAA CTG C-3′
COL2 A1	Primer forward (5′-3′)	5′-AAG AAA CAC ATC TGG TTT GGA GAA A-3′
	Primer reverse (5′-3′)	5′-TGG GAG CCA GGT TGT CAT C-3′
	Probe (5′FAM/3′TAMRA)	5′-CAA CGG TGG CTT CCA CTT CAG CTA TGG-3′
IL-1β	Primer forward (5′-3′)	5′-TTA CTA CAG TGA CGA GAA TGA GCT GTT-3′
	Primer reverse (5′-3′)	5′-GGT CCA GGT GTT GGA TGC A-3′
	Probe (5′FAM/3′TAMRA)	5′-CTC TTC ATC TGT TTA GGG TCA TCA GCC TCA A-3′
RPLP0		Bt03218086_m1
ELN		Bt03216594_m1
IL-8		Bt03211906_m1
MCAM		Bt03258894_m1
MKX		Bt04292311_m1
MMP3		Bt04259490_m1
TAGLN		Bt03234600_m1
ADAMTS5		Bt04230789_m1

## Statistics

GraphPad Prism 9 (version 9.3.1, GraphPad Software, LLC, San Diego, United States) was used for statistical analysis of the data. Data were not normally distributed as defined by Shapiro-wilk normality test. Kruskal Wallis with Dunn’s post-hoc test was used to determine differences among experimental groups. Values of *p* < 0.05 (*), *p* < 0.01 (**), *p* < 0.001 (***), and *p* < 0.0001 (****) were considered statistically significant.

## Results

### Macroscopic IVD changes with chemonucleolysis

**FIGURE 2:**
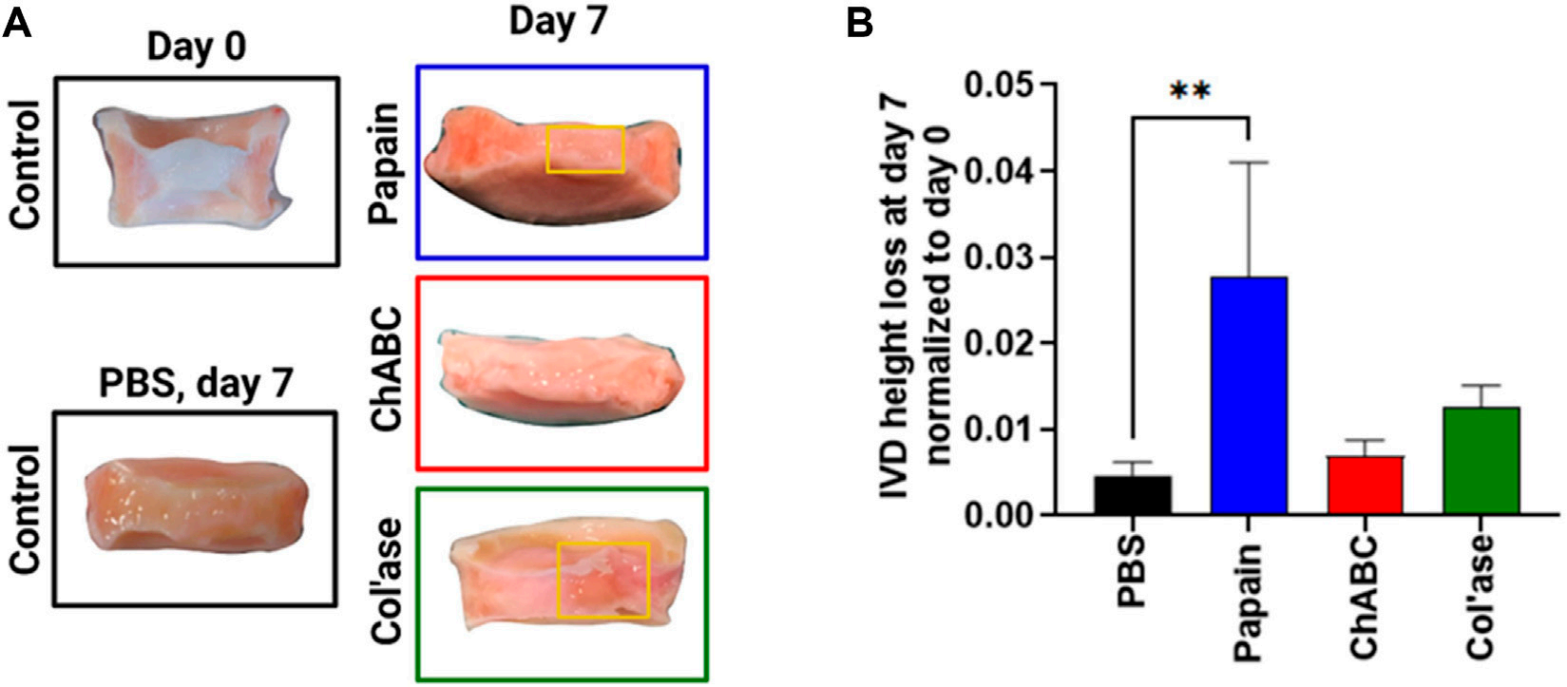
**(A)** Gross morphology of the IVD study groups. At day 7 post-enzyme treatment, macroscopic voids were observed in the papain and col’ase-injected specimens, indicated by the yellow boxes, but not in chABC-injected specimens. **(B)** Height loss during 7 days of IVD culture with daily loading in the bioreactor, calculated relative to the initial IVD height after dissection. Papain produced the highest magnitude height loss in the IVD specimens compared to the PBS control. Data are shown as the mean and standard deviation of *n* = 4 replicates, where ** indicates *p* = 0.0065.

Differences between the enzyme treatments were evident in the gross morphology of the IVD specimens shown in Figure 2A. Papain and col’ase produced macroscopic tissue voids at the center of the IVDs in the region local to enzyme injection (yellow boxes), but no macroscopic voids were observed in the chABC samples.

The IVD height change at day 7 of culture period was calculated and normalized to initial height after dissection (day 0) (Figure 2B). Papain digestion induced the greatest height loss compared to day 0 (nearly 3%), whereas chABC and col’ase induced approximately 0.6% and 1.2% height loss. All three enzyme treatments induced greater height loss than the PBS control, with difference between papain and PBS (*p* = 0.0065) being statistically significant.

### GAG content changes with chemonucleolysis

**FIGURE 3:**
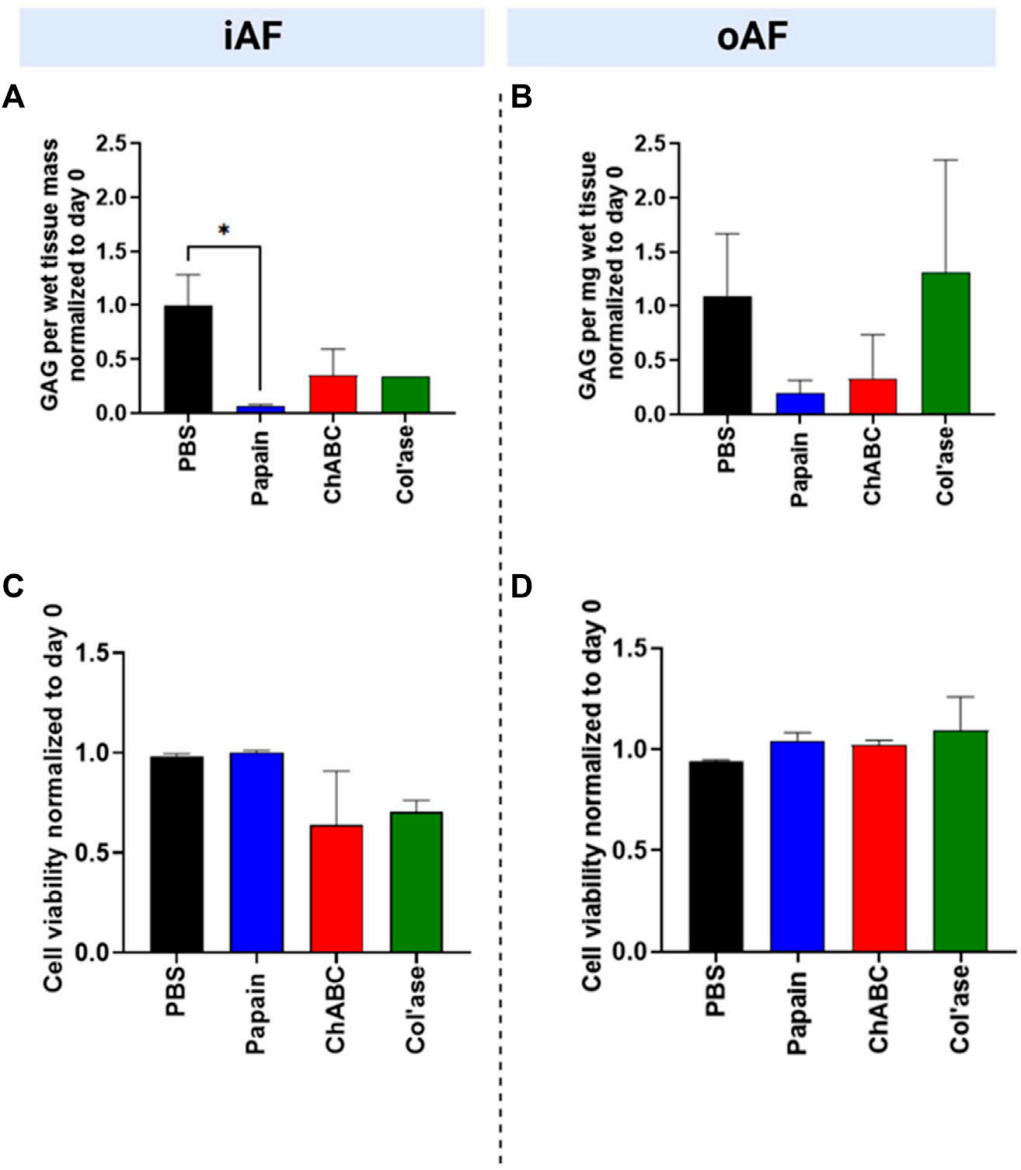
Biochemical and cellular changes in the IVDs at day 7 after enzyme treatment. GAG content/wet tissue mass of **(A)** iAF and **(B)** oAF tissue samples at day 7 normalized to day 0 of the respective donor. Papain produced the largest drop in GAG content of the iAF and oAF compared to the PBS control, the drop being statistically significant for the iAF (*p* = 0.0143.) **(C)** Cell viability (%) at day 7 normalized to day 0 of the respective donor for the iAF and **(D)** oAF of the IVDs quantified from LDH staining images. No significant changes in cell viability were measured compared to the PBS control in the iAF or oAF, although cell viability in the iAF for the papain-treated samples trended highest (close to 100%) compared to the chABC and col’ase (60%–70%). Results are shown as the mean and standard deviation of four replicates, except for **(A)** col’ase, where one replicate could only be recovered due to digestion.

The GAG content of the tissues relative to day 0 is shown in Figure 3. In the iAF, the PBS control samples retained approximately 100% of the GAG content compared to day 0. In contrast, papain digested tissue was measured to retain less than 10% of the GAG content relative to day 0. ChABC and col’ase each exhibited approximately 35% retention of GAG content relative to day 0. The change in iAF GAG content measured over the culture period compared to the PBS-injected control was only significant for papain (*p* = 0.0143, Figure 3A). In the oAF (Figure 3B), papain and chABC digestion tended to decrease GAG content of the tissues, although the shifts were not statistically significant compared to the PBS control (*p* > 0.05). The col’ase group tended to increase GAG content, but the differences among the groups and in comparison to the PBS control were not statistically significant (*p* > 0.05).

### Cell viability after chemonucleolysis

In the iAF, adjacent to tissue void, papain digestion produced no changes in cell viability relative to day 0 (Figure 3C). For all papain samples, cell viability remained at nearly 100% at day 7 of culture. Compared to papain, chABC and Col’ase treatment produced decreasing trends in cell viability in the iAF to approximately 60%–70% at day 7 culture, though the shifts were not statistically significant compared to PBS (*p* > 0.05). Cell viability in the oAF was maintained at nearly 100% for the duration of the culture period for all sample groups, with no statistical differences detected between PBS-injected and any of the enzyme-injected groups (*p* > 0.05, Figure 3D).

### Histological changes with chemonucleolysis

**FIGURE 4:**
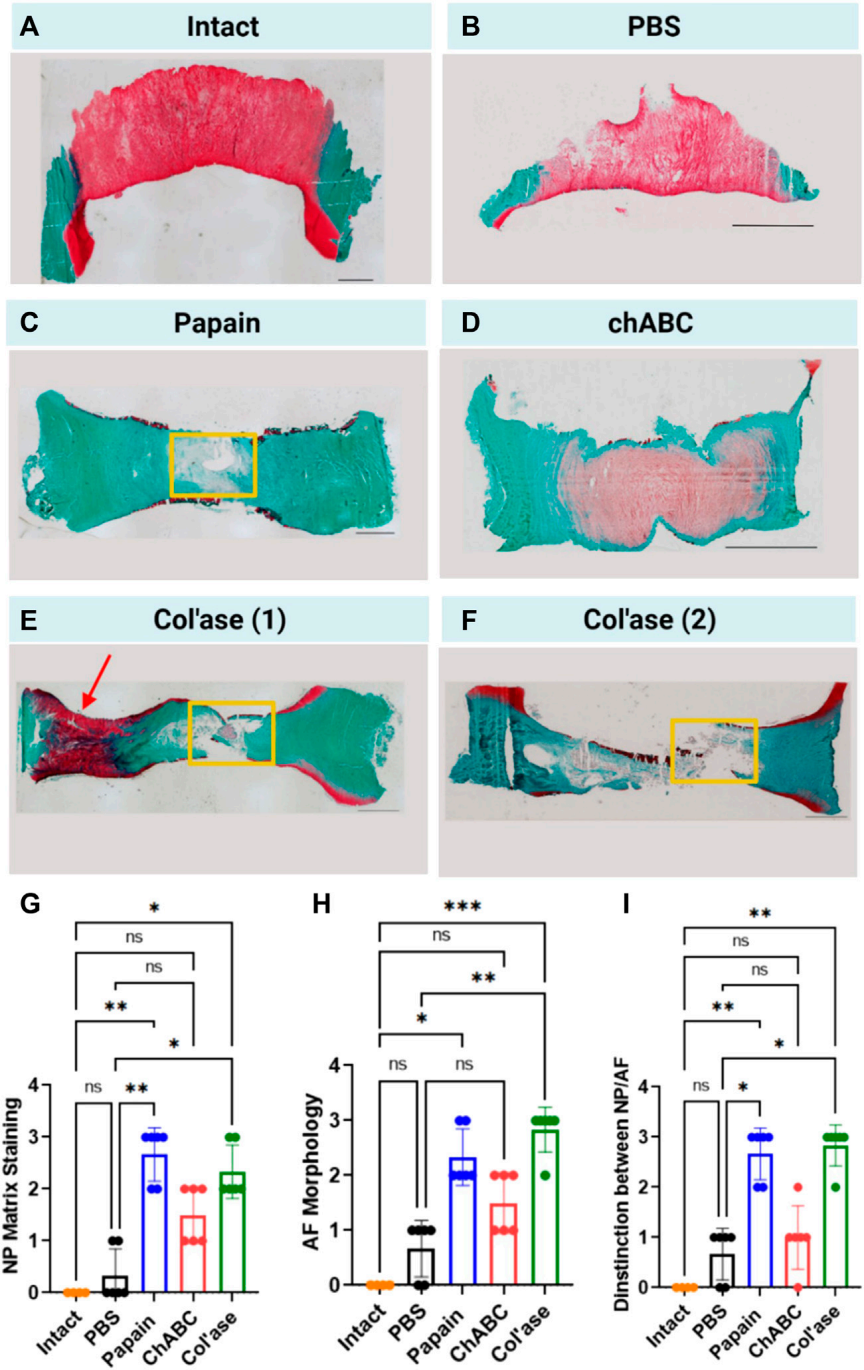
Safranin-O and fast green staining across whole sagittal cross-sectional areas of the IVD sample groups highlighting the morphology of GAG (red) and collagens (green) in the matrix for **(A)** Day 0 intact control, and at day 7 for **(B)** PBS control, **(C)** papain, **(D)** chABC, **(E)** col’ase, specimen 1, and **(F)** col’ase, specimen 2. While voids and significant GAG loss could be identified at day 7 in papain and col’ase treated specimens, chABC produced the mildest changes, with no void produced and some GAG staining remaining. Two representative specimens are shown for the col’ase group to demonstrate a region of concentrated GAG staining observed in the oAF of one of the samples (red arrow). Yellow boxes highlight macroscopic voids produced by enzyme digestion. Scale bars = 2 mm. **(G,H,I)** Histological scoring values based on criteria detailed in Table 1 for Safranin O/Fast green staining of whole sagittal cross-sections. Overall, papain and col’ase produced higher degeneration scores than chABC, PBS and day 0 intact controls. Grading was assessed by two blinded observers on three sections per group, where *p < 0.05, **p < 0.01, and ***p < 0.001.

Shown in Figure 4 are representative overview histological images of safranin-O and fast green staining to highlight the distributions of GAG and collagens, respectively. PBS-injected controls exhibited preservation of GAG staining in the NP region at day 7 of culture (Figure 4B) compared to day 0 (Figure 4A). Papain digestion resulted in complete obliteration of the GAG staining across the sagittal cross-sectional area of the IVDs (Figure 4C), but left behind collagenous tissues, evidenced by fast green staining. Papain also induced the formation of millimeter-scale voids containing small tissue fragments (Figure 4C, yellow box). Conversely, no voids of similar scale were observed after chABC treatment (Figure 4D). Col’ase produced voids similar in size to papain (Figures 4E, F, yellow box). One of the specimens in the collagenase group exhibited a region of highly concentrated GAG staining in the oAF (Figure 4E, red arrow).

A modified histological scoring standard was used based on literature (Lee et al., 2021) to assess degeneration from the histological overviews. The scoring standard allowed for an assessment of proteoglycan staining in the NP (Figure 4G), morphological organization of the AF (Figure 4H), and the structural distinction between the NP and AF (Figure 4I) at day 7 for the PBS and enzyme-treated groups compared to day 0 intact. Across all grading criteria, no significant differences in degeneration scores between PBS and day 0 intact were measured (*p* > 0.05). Additionally, chABC treatment induced no significant changes in degeneration score at day 7 (*p* > 0.05). In contrast, papain and col’ase enzyme-treated samples exhibited similar grades of degeneration (*p* > 0.05). Also, papain and col’ase treatment produced statistically significant increases in degeneration grade compared to the PBS-inject controls at day 7 and day 0 intact samples (*p* < 0.05).

**FIGURE 5:**
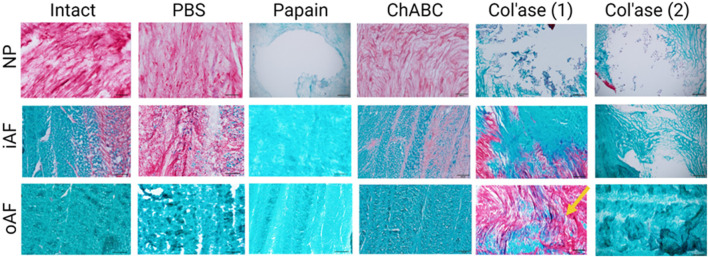
High magnification images of Safranin-O and fast green staining highlighting the morphology of GAG (red) and collagens (green) in the nucleus pulposus (NP), inner annulus fibrosus (iAF) and outer annulus fibrosus (oAF) of the intact (day 0), PBS-treated (day 7), and enzyme-treated (day 7) groups. Overall, papain and col’ase digestion resulted in complete loss of GAG staining in the NP region. GAG staining could still be identified in the NP and iAF of chABC treated specimens. Two representative specimens are shown for the col’ase group to demonstrate a region of concentrated GAG staining observed in the oAF of one of the samples (yellow arrow). Scale bars = 200 µm.

High magnification images of the NP, iAF and oAF regions of each of these specimens are shown in Figure 5.

### Phenotypic characterization after chemonucleolysis

**FIGURE 6:**
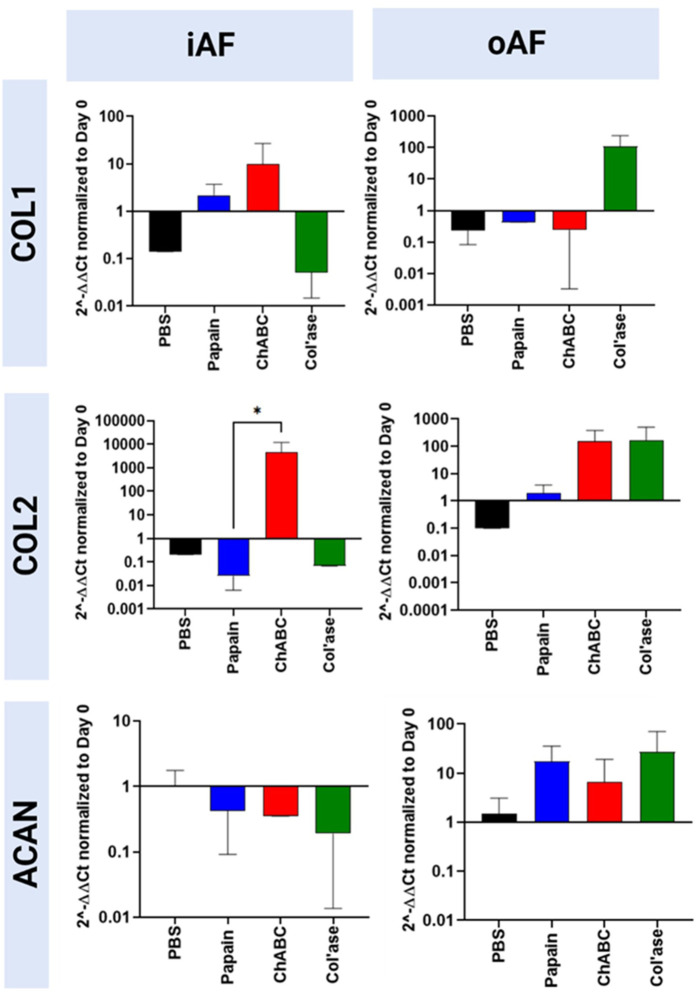
Bovine mRNA expression at day 7 for the major IVD ECM components, COL1, COL2 and ACAN, relative to day 0 and RPLP0 for the PBS-injected and enzyme-treated sample groups. In the iAF, papain and col’ase resulted in mild downregulation in the major disc NP matrix markers, ACAN and COL2. ChABC treatment resulted in a statistically significant upregulation of a major ECM marker, COL2, within the iAF compared to papain (*p = 0.0286), suggesting chABC could be inducing the mildest degenerative response. Results are shown as the mean and standard deviation of four replicates.

Gene expression for the major disc ECM markers, COL1, COL2 and ACAN, in the iAF and oAF of the treatment groups is shown in Figure 6. ChABC digestion resulted in increased trends in gene expression for both COL1 and COL2 in the iAF of loaded specimens compared to PBS controls, though the differences were not statistically significant (*p* > 0.05). An upregulation in COL2 gene expression was measured for the chABC group that was significantly higher than that of the papain group (*p* = 0.0286). ACAN expression tended to be downregulated with all enzyme treatments compared to PBS (*p* > 0.05). In the oAF, col’ase digestion produced increasing trends in gene expression for COL1, COL2 and ACAN (*p* > 0.05). ChABC injection tended to increase COL2 and aggrecan expression (*p* > 0.05).

**FIGURE 7:**
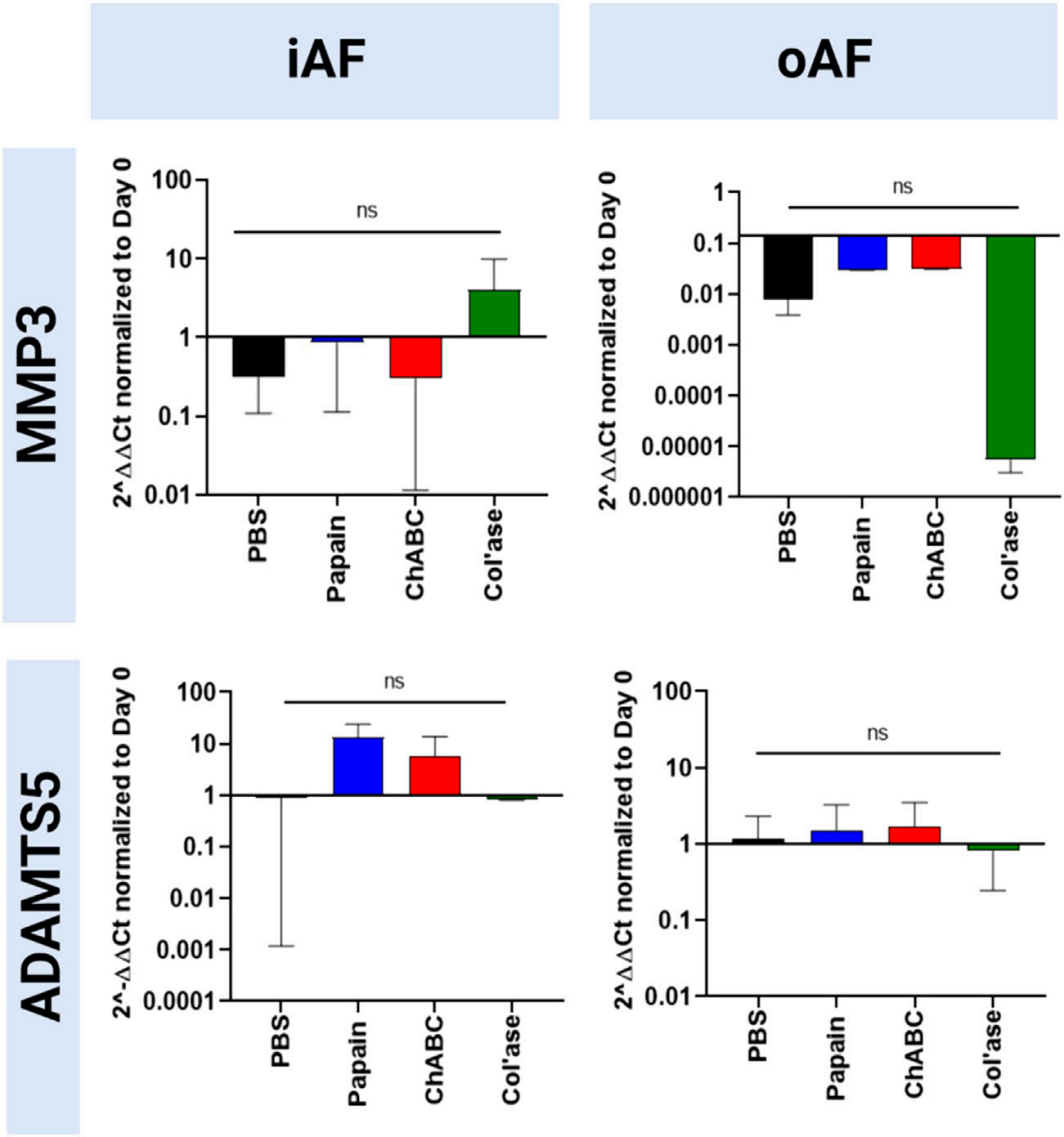
Bovine mRNA expression at day 7 for catabolic enzymes, ADAMTS5 and MMP3, relative to day 0 and RPLP0 for the PBS-injected and enzyme-treated sample groups. The highest magnitude shift was observed for MMP3 in the oAF, although no statistically significant shifts were detected (*p* > 0.05). Results are shown as the mean and standard deviation of four replicates.

Gene expression for catabolic enzymes MMP3 and ADAMTS5 are shown in Figure 7. In the iAF, col’ase treatment produced a small increase in gene expression for MMP3 relative to PBS control (*p* > 0.05). Papain and chABC produced small increasing trends in ADAMTS5 expression compared to the PBS control (*p* > 0.05). In the oAF, general trends of decreasing MMP3 expression were observed, though the shifts were not significant (*p* > 0.05). ADAMTS5 expression was largely unchanged in oAF with enzyme or PBS injection (*p* > 0.05)

**FIGURE 8:**
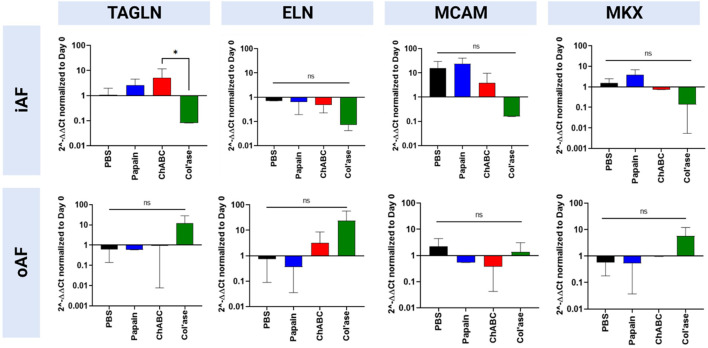
Bovine mRNA expression at day 7 for oAF markers, TAGLN, ELN, MCAM, and MKX, relative to day 0 and RPLP0 for the PBS-injected and enzyme-treated sample groups. Notably, in the iAF, col’ase treatment tended to downregulate expression of all oAF markers compared to the PBS control, although the shifts were not statistically significant (*p* > 0.05). In the oAF, col’ase treatment tended to upregulate oAF markers compared to PBS (*p* > 0.05). Results are shown as the mean and standard deviation of four replicates with *p = 0.0360.

Expression of oAF-specific markers (MCAM, ELN, TAGLN and MKX) are shown in Figure 8. In the iAF, col’ase treatment tended to downregulate expression of oAF markers, TAGLN, ELN, MCAM and MKX, compared to the PBS control (*p* > 0.05). Relative gene expression for TAGLN was significantly higher in the chABC group compared to col’ase (*p* = 0.0360). Papain and chABC tended to upregulate TAGLN and MKX relative to the PBS control (*p* > 0.05). In the oAF, no significant shifts in gene expression for MCAM, ELN, TAGLN or MKX were measured relative the PBS controls (*p* > 0.05). However, the expression of ELN, TAGLN and MKX tended to increase with col’ase injection relative to the PBS control.

**FIGURE 9:**
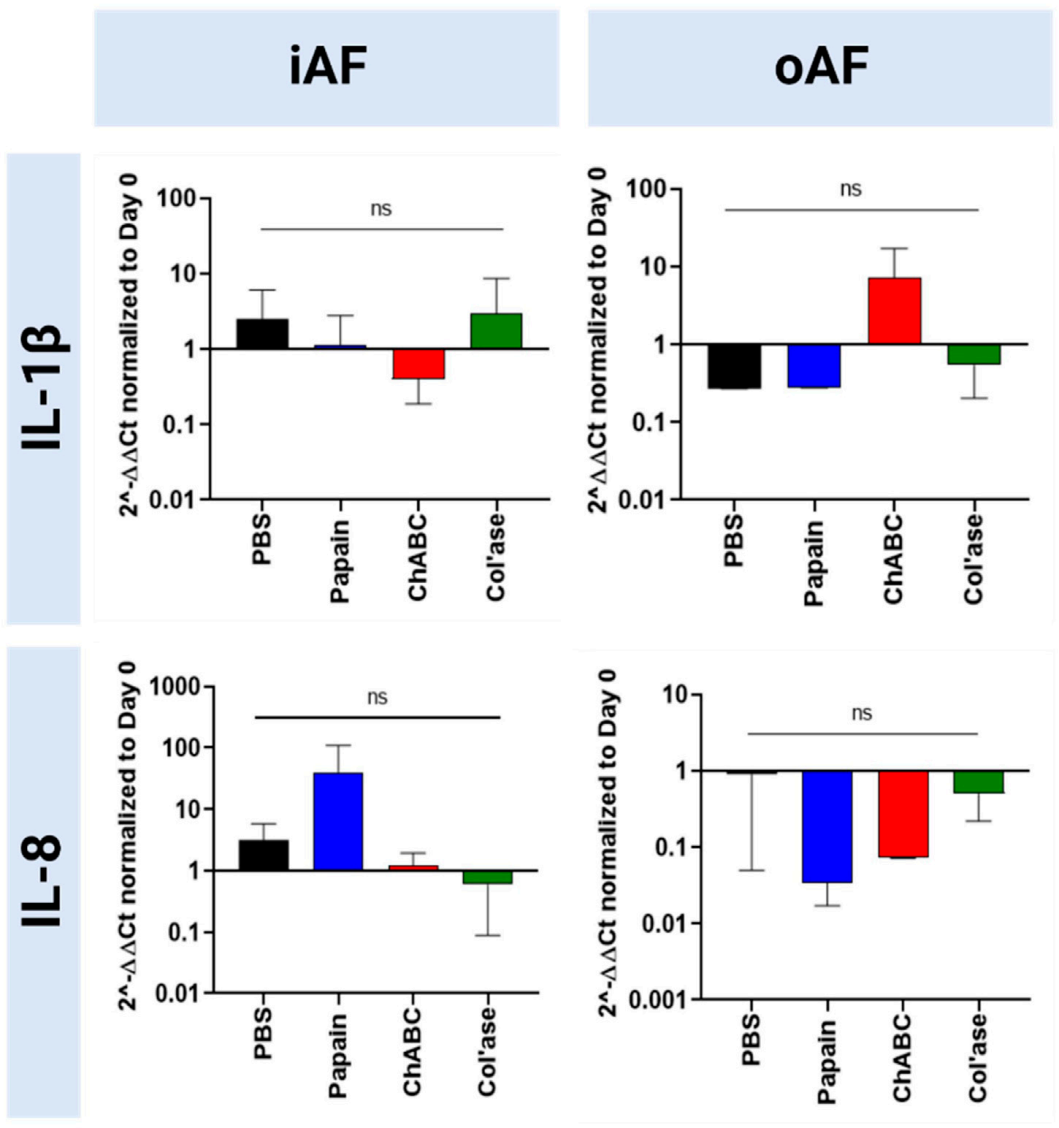
Bovine mRNA expression at day 7 for proinflammatory markers relative to day 0 and RPLP0 for the PBS-injected and enzyme-treated sample groups. Papain treatment produced the highest upregulation in the expression of a proinflammatory marker, 1L-1β, although the none of the shifts were statistically significant relative to each other or the PBS control (*p* > 0.05). Results are shown as the mean and standard deviation of four replicates.

Expression of pro-inflammatory markers (IL-1β and IL-8) is shown in Figure 9. In the iAF, there were no significant shifts in IL-1β expression compared to the PBS control (*p* > 0.05). Relative gene expression for IL-8 tended towards upregulation with papain, yet the change was not statistically significant compared to the PBS control (*p* > 0.05). In the oAF, no significant shifts in gene expression for IL-1β or IL-8 were measured relative to PBS controls (*p* > 0.05). However, compared to PBS controls, chABC tended to increase the expression of IL-1β, and both papain and chABC tended to decrease the expression of IL-8 (*p* > 0.05).

## Discussion

The aim of this study was to develop and compare three different *ex vivo* bovine organ culture models of chemonucleolysis-induced IVD degeneration. *Ex vivo* models, which bridge *in vitro* and *in vivo* studies, are essential tools for evaluating therapeutic interventions for IVD degeneration by providing a cost-effective route for recapitulating the multifactorial 3D microenvironment of the IVD. With increasing relevance of bioreactor-loaded whole organ cultures (Pfannkuche et al., 2020), the field of IVD repair needs more *ex vivo* models, providing researchers with a robust set of pre-clinical evaluation tools to utilize for thorough examination of therapeutic efficacy. To this end, we aimed to deepen the understanding of IVD chemonucleolysis by targeting moderate levels of degeneration with three different enzymes, papain, chABC and col’ase and comparing the effects side-by-side. It was hypothesized that the distinct mechanisms of ECM digestion from each of the enzymes would produce differing degenerative effects on the explanted tissues, helping to capture more aspects of human pathogenesis.

Notably, in prior *ex vivo* studies with papain and trypsin, enzymatic digestion alone was not sufficient to induce dimensional changes in the IVDs (Chan et al., 2013). In this study, application of enzymes combined with daily dynamic loading resulted in IVD height loss compared to the PBS treated samples. Height loss was most pronounced for the papain-treated samples. IVD height loss can be regarded as the result of decreased mechanical integrity of the ECM (Balkovec et al., 2013; Yoganandan et al., 2017). Aggrecan molecules are known to confer mechanical properties, like compressibility and elasticity, to the ECM through stabilizing intermolecular interactions (Pratta et al., 2003). In the decreased presence of proteoglycan, which was most extensive after papain treatment, extrinsic loading allowed mechanical fatigue of the remaining tissues, resulting in dimensional changes such as height loss.

The differences in substrate specificity among the three enzymes used in the study made it impractical to target a single concentration for their application as chemonucleolytic agents. Rather, each enzyme was applied in a concentration shown to induce moderate degeneration in prior *in vivo* and *ex vivo* IVD studies (Chan et al., 2013; Gullbrand et al., 2017; Rustenburg et al., 2020). All enzymes induced an overall loss of GAG in the iAF region, with papain having the most pronounced effect and producing voids around the site of injection. With its known proteolytic activity towards polypeptides (Mamboya, 2012), papain does not directly degrade GAG side chains. However, GAG loss is associated with the enzyme in this and prior studies (Roberts et al., 2008; Chan et al., 2013), suggesting papain activity for cleaving the link protein or core protein on aggrecan. Conversely, chABC cleaves chondroitin 4-sulfate, dermatan sulfate, and chondroitin 6-sulfate side chains on aggrecan molecules (Takagaki and Kakizaki, 2007). In our study, this enzyme produced milder losses of GAG in the NP and iAF compared to papain and no major voids. The col’ase class II enzyme, with broad specificity to multiple types of native collagen fibrils (Van Wart and Steinbrink, 1985), produced tissue voids similar in size to papain. The formation of a void, which implies loss of both GAG and collagen induced by col’ase, can be explained by the fact that aggrecan molecules interact with the collagen network (Adams et al., 1977; Zhou et al., 2021). Thus, release of GAG from the tissues may occur concomitantly with collagen. Generally, matrix degradation and void formation are important considerations for evaluating NP replacement or repair materials. Biomaterial implantation may require ECM removal to relieve intradiscal pressures and provide physical space for accommodation (Roberts et al., 2008). Also, void-producing chemonucleolytic models provide proximity to the current clinical practice of discectomy. Within this context, the papain and col’ase models provide more functionality than chABC. Conversely, the chABC model may represent structural similarity with early stages of degeneration.

A goal of this study was to compare gene expression changes after chemonucleolysis to understand the extent to which the models mimic the phenotypical changes in natural human IVD degeneration. Generally, none of the enzymes used in our study induced gene expression changes exactly following reported degenerative patterns for iAF tissue, which generally involves concomitant upregulation of type I collagen (Le Maitre et al., 2007a; Markova et al., 2013), downregulation of collagen II and proteoglycan (Le Maitre et al., 2007a; Lian et al., 2017; Silagi et al., 2018), and upregulation of matrix degrading enzymes like MMP3 (Roberts et al., 2000; Le Maitre et al., 2004) and ADAMTS5 (Gruber et al., 2007; Pockert et al., 2009). Interestingly, chABC significantly upregulated COL2 expression in the iAF, which could be indicative of a compensatory behavior by the IVD cells, making it a potential model for early-stage degeneration (Cs-Szabo et al., 2002; Uei et al., 2006; Markova et al., 2013; Malonzo et al., 2015). Whereas, papain and col’ase provided no such upregulation, and thus in this respect, more closely following advanced degeneration. Interestingly, enzyme digestion did not induce a profound proinflammatory effect, as indicated by no significant shifts measured in IL-1β or IL-8 gene expression, an outcome also reported in a previous organ culture model of degeneration induced by one strike loading (Zhou et al., 2021).

Throughout the 7-day culture period, no major cracks or fissures were formed in the oAF with chemonucleolysis. It is possible that more prolonged culture conditions, especially in combination with loading, would lead to formation of severe structural abnormalities in the oAF region. Worth noting is the effect of enzyme digestion on GAG content in the oAF. Tissue histology detected an area of concentrated GAG staining for one of the col’ase -digested specimens. Possibly, this was a rescue effect by oAF cells, which in prior reports have been shown to retain capacity for repair during IVD degeneration resulting in an upregulation of proteoglycan (Cs-Szabo et al., 2002). Further indicative of a “rescue” capacity of the host IVD cells in the oAF, anabolic trends in mRNA expression were detected for COL2, ACAN and COL1 with col’ase injection.

Taking a further look at cell scale responses, viability remained largely unaffected in the oAF after enzyme treatment. In the iAF, on the other hand, cell viability depended on enzyme treatment, with the papain-digested specimens retaining higher cell viability than chABC and col’ase-injected groups. A recent study used attenuated total reflectance Fourier-transform infrared spectroscopy to demonstrate the structural soundness of collagen networks following papain digestion (Silva et al., 2016). Differences in nanoscale tissue organization and conformational properties of adhesion domains on collagens and aggrecan post-chemonucleolysis is a possible factor that could account for the higher cell viability observed with papain versus the other enzymes, and is an area requiring further study. Human IVD degeneration and acute tissue injury have been reported to be associated with decreases in cell viability and metabolic activity (Iatridis et al., 2009; Alkhatib et al., 2014). It stands to reason that the papain model, which leaves cell viability unaffected, may yield regenerative responses more predictive of clinical outcomes more representative for trauma or nucleotomy cases. IVD degeneration has also been marked by increased clustering and proliferation (Johnson et al., 2001), effects which were not observed by histology. Thus, the enzyme models presented in this study may replicate some, but not all, instances of abnormal cellular activity occurring during degeneration.

This study aimed to enhance our understanding of chemonucleolytic *ex vivo* models of IVD degeneration. Our results demonstrate that all three enzymes induced distinct changes in the major categories associated with clinical disc degeneration, including histology, IVD height loss, cell viability, and gene expression. Analyzing the outcomes of each enzyme, as summarized in Table 3, we can generally conclude that chABC most closely resembles the early stages of degeneration compared to the other enzymes. Yet, it is important to note that a single chemonucleolysis model is unlikely to replicate all the complex pathological features present in *in vivo* lumbar IVD degeneration. This work significantly contributes to the understanding of *ex vivo* bovine models of chemonucleolysis by comparing outcomes of three enzymes side by side, and we posit that all three models have potential to serve as viable models for evaluating the efficacy of therapeutic approaches under development. Furthermore, we propose that studying therapeutic outcomes with all three models can aid in providing a comprehensive evaluation of regenerative outcomes.

**TABLE 3:** Summary of the major changes observed in bovine caudal intervertebral discs induced by chemonucleolysis combined with daily dynamic loading.

Enzyme	Dose (U/mL)	Void?	Height loss (%)	Histology	Cell viability	Gene expression adjacent to injection site
Papain	65	Yes	Highest (2.77% ± 1.3%)	Complete loss of GAG staining in NP, high degeneration scores	100% ± 1.1% in iAF, 100% ± 3.7% in oAF	Mild downregulation in matrix markers (COL2, ACAN), mild upregulation ADAMTS5 and IL-1β
chABC	5	No	Lowest (0.69% ± .2%)	Some GAG staining remaining in NP, lower degeneration scores	64% ± 27.0% in iAF, 100% ± 2.1% oAF	Moderate upregulation in COL2, mild upregulation in COL1 and ADAMTS5
Col’ase	0.5	Yes	Intermediate (1.25% ± .2%)	Complete loss of GAG staining in NP; concentrated region observed in oAF for one sample, high degeneration scores	70.3% ± 5.9% in iAF, 100% ± 16.4% in oAF	Mild downregulation in matrix markers (COL1, COL2, ACAN) and mild upregulation MMP3

However, limitations of this study are to be noted. Morphology of tissue voids may be produced more uniform with an automated, rather than manual, enzyme injection process, making the degenerative changes potentially more reproducible. Gene expression of neurotropic factors, such as brain derived neurotropic factor (BDNF), and angiogenic factors, such as VEGF, are important to evaluate the models' predictive capacity for pain response *in vivo* and should be evaluated in future studies. Finally, spatiotemporal transcriptomic and proteomic profiling should be applied in the tissue models, towards developing a more comprehensive understanding of tissue response to enzymatic degradation and the extent to which it mimics human degeneration.

## Conclusion

We did a side-by-side comparison of the effects of chemonucleolysis on bovine coccygeal IVDs by three different enzymes, papain, chABC, and col’ase after 7 days of loaded culture. Generally, all three enzymes were useful for recapitulating certain clinically and biologically relevant aspects of human IVD degeneration. Papain produced macroscopic tissue voids and the most aggressive GAG and height loss, although cell viability in the remaining tissues was largely preserved. In contrast to this, ChABC and col’ase digestion caused greater losses in cell viability. Compared to papain, col’ase produced similar size tissue voids. Because chABC did not produce macroscopic tissue voids, but the mildest height loss and an anabolic shift in iAF COL2 expression, it is concluded that chABC recapitulates early-stage degeneration more so than papain and col’ase. This work significantly contributes to the understanding of *ex vivo* bovine models of chemonucleolysis by comparing outcomes of three enzymes side by side. Overall, the results of our study show that chemonucleolysis is a useful tool providing researchers with a robust spectrum of degenerative changes in IVD tissues and the models have potential as tools for assessment of therapeutic interventions.

## Data Availability

The original contributions presented in the study are included in the article/Supplementary Material, further inquiries can be directed to the corresponding author.
